# Development and Optimization of Polymer-Based Dissolving Microneedles Fabricated by Mold Casting Method

**DOI:** 10.3390/polym18101255

**Published:** 2026-05-21

**Authors:** Liubov Bodnar, Tetiana Kovalova, Volodymyr Yakovenko, Oleh Koshovyi, Kaloyan D. Georgiev, Iliya Zhelev Slavov, Liliia Vyshnevska

**Affiliations:** 1 Drug Technology Department, Pharmaceutical Faculty, National University of Pharmacy, St. Hryhoriya Skovorody, 53, 61002 Kharkiv, Ukraine; bodnar_la@ukr.net (L.B.); tatyko72@gmail.com (T.K.); 2 Institute for the Further Training of Pharmacy Professionals, National University of Pharmacy, St. Hryhoriya Skovorody, 53, 61002 Kharkiv, Ukraine; v.iakovenko@gmail.com; 3 Institute of Pharmacy, University of Tartu, Nooruse Str., 1, 50411 Tartu, Estonia; 4 Department of Clinical Pharmacy, Pharmacotherapy, Pharmacognosy and Pharmaceutical Chemistry, Zaporizhzhia State Medical and Pharmaceutical University, 26 Marii Prymachenko Blvd., 69035 Zaporizhzhia, Ukraine; 5 Department of Pharmacology, Toxicology and Pharmacotherapy, Faculty of Pharmacy, Medical University of Varna, 9000 Varna, Bulgaria; kaloyan.georgiev@mu-varna.bg; 6 Department of Biology, Faculty of Pharmacy, Medical University of Varna, 9000 Varna, Bulgaria; ilia.slavov@mu-varna.bg

**Keywords:** microneedles, 3D printing, transdermal systems, mold casting, polymer base

## Abstract

Microneedle systems represent a promising minimally invasive approach for transdermal drug delivery; however, their performance strongly depends on the composition and mechanical properties of the polymer matrix. The aim of this study was to select an optimal polymer composition for the fabrication of dissolving microneedle arrays produced by the mold casting method. The study focused on evaluating mechanical strength, dissolution behavior, and penetration efficiency of different polymer systems. Microneedle matrices were fabricated using polyvinylpyrrolidone (PVP K-30), methylcellulose, sodium alginate, and hyaluronic acid at various concentrations, alone and in combination. No active pharmaceutical ingredient (API) was incorporated; the study was performed using blank polymeric systems intended for subsequent drug loading. The microneedles were manufactured using 3D-printed and silicone molds. Their performance was evaluated by in vitro dissolution testing, pH measurement, penetration studies in gelatin gel and Parafilm M models, and mechanical compression testing. Monopolymer systems demonstrated either rapid dissolution with insufficient mechanical strength or improved strength at the expense of prolonged dissolution time. Combined polymer formulations showed superior structural uniformity and balanced performance. In particular, the system containing 5% PVP K-30 and 10% sodium alginate demonstrated the best overall characteristics, achieving high penetration efficiency (up to 96%), uniform dissolution (78%), and appropriate dissolution time (8.5 ± 0.5 min). Addition of hyaluronic acid further improved structural uniformity and handling properties. The results indicate that composite polymer matrices provide an optimal balance between mechanical stability, penetration ability, and dissolution rate. The formulation consisting of 5% PVP K-30 and 10% sodium alginate was identified as the most promising base for further development of drug-loaded dissolving microneedle systems.

## 1. Introduction

**Figure 1:**
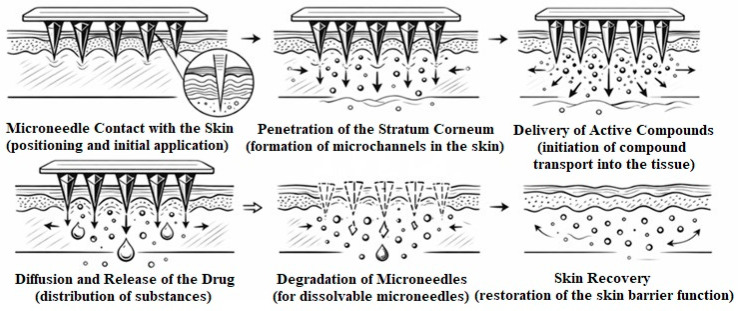
Principle of microneedle action.

Traditional methods of substance administration in the body are often associated with pain and discomfort due to injections, as well as with the risk of infectious complications and the need for medical personnel. In this context, microneedle systems are considered a promising approach for transdermal delivery, providing a minimally invasive and painless administration method. Their mechanism of action is based on the creation of microchannels in the stratum corneum of the epidermis without reaching the deeper layers of the dermis, where most nociceptors and blood vessels are located (Figure 1). This allows temporary overcoming of the skin’s barrier function and ensures controlled diffusion or release of active components into the intercellular space [1,2,3]. In this study, the performance of microneedle systems is primarily evaluated in terms of how the composition and concentration of the polymer matrix influence key functional properties, including mechanical stability, structural integrity, and the controlled formation of microchannels in barrier-mimicking membranes. Unlike general descriptions of transdermal transport mechanisms, which are well established in the literature, the present work focuses on formulation-dependent behavior and its direct impact on microneedle fabrication quality and functional efficiency. In particular, emphasis is placed on the relationship between polymer network structure, mechanical resistance during insertion, and subsequent dissolution dynamics, which collectively determine the overall applicability of the developed systems for transdermal delivery.

Microneedles enable precise delivery of components to a specified depth of the skin, ensuring effective interaction with local cells and the tissue microenvironment. Depending on the type of system (solid, hollow, coated, or dissolvable), the transport mechanism can occur through pre-formed microchannels followed by drug application, direct solution delivery through the needle lumen, or controlled release of encapsulated substances during degradation of the polymer matrix. Additionally, micromechanical stimulation of tissues induces a local biological response, including cell activation and enhanced regenerative processes, which broadens the functional potential of this drug delivery method [4,5,6,7].

The main advantages of microneedles include painlessness and reduced psychological barriers for patients, high delivery precision, the possibility of lowering therapeutic doses due to localized action, minimization of systemic toxicity, and reduced risk of infection compared to traditional injection methods. Microneedle systems can also maintain the stability of biologically active molecules, including proteins and vaccines, and potentially allow self-administration without specialized equipment or personnel [8,9,10,11,12].

**Figure 2:**
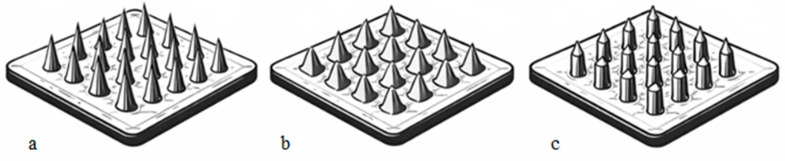
Shapes of microneedle systems ((**a**) conical; (**b**) pyramidal; and (**c**) cylindrical).

Microneedles are classified by shape (conical, pyramidal, rod-shaped, Figure 2), material (dissolvable, non-dissolvable), method of use (single-use, reusable), and purpose (transdermal drug delivery, cosmetic procedures, local tissue stimulation). Each type has specific mechanical properties and penetration characteristics that affect both efficacy and safety.

The goal of contemporary research is to develop microneedle systems with optimal mechanical properties, high penetration efficiency, biocompatibility, and stability, opening new possibilities for the advancement of transdermal technologies and personalized medicine. In this study, “optimal” microneedle formulation was defined based on a combination of penetration efficiency ≥ 80%, complete dissolution time within 5–15 min, and structural integrity without visible deformation after demolding. For example, Aziz A. et al. demonstrated that combining microneedles with enhancers in a transdermal patch significantly improves the penetration of bisoprolol fumarate through the skin, creating microchannels that facilitate faster and greater drug delivery for potential blood pressure control with minimal skin irritation [13].

Abuershaid J. et al. investigated microneedles made from a copolymer of lactic and glycolic acids loaded with minocycline for acne treatment. They found that these microneedles effectively penetrate the skin, dissolve rapidly, and provide substantial local drug deposition with strong antibacterial activity [14].

Zeng W. et al. developed a microneedle patch containing cryptotanshinone that responds to excess reactive oxygen species in infected wounds, enabling controlled drug release while simultaneously providing antibacterial and antioxidant effects to improve healing [15].

Saraswat P. et al. worked on the development and optimization of a biodegradable polymeric microneedle patch with vitamin B12 to enhance penetration and achieve effective release of this water-soluble vitamin during transdermal delivery [16].

In modern microdelivery research, high accuracy and reproducibility of microneedle characteristics are critical. Fabrication of microneedles using mold casting allows for controlled geometry, needle height, and sharpness while ensuring standardization of the manufacturing process. This method is an effective approach to obtain microneedles with defined parameters, confirming its relevance for both fundamental and applied studies [17].

The objective of this study was to develop and fabricate dissolving microneedle arrays using the mold casting method, as well as to systematically investigate the influence of polymer composition and concentration on their key functional properties, including mechanical strength, dissolution behavior, and penetration efficiency in barrier-mimicking models.

## 2. Materials and Methods

### 2.1. Materials

For the fabrication of the microneedle matrix, the following were used: polyvinylpyrrolidone (PVP K-30, Fengchen Group, Qingdao, China), methylcellulose (Shandong Landu New Material Co., Ltd., Jinan, China), sodium alginate (No. 20230506C, Nane, Shenzhen, China), hyaluronic acid (No. 24050401, Shenzhen Unison Bio-tech Co., Ltd., Shenzhen, China), and purified water prepared under laboratory conditions. For penetration studies, medical gelatin (Gelken Gelatin, Xiamen, China) was used. All substances were of analytical grade and used without further purification [18,19,20,21,22,23].

### 2.2. Methods

#### 2.2.1. Visual Inspection

Visual control was performed immediately after removal from the mold, as well as after penetration and dissolution tests. The uniformity of needle formation; tip sharpness; and presence or absence of air bubbles, cracks, or deformations were assessed.

#### 2.2.2. pH Determination

A 0.1 g sample of microneedles, ground to a homogeneous mass in a mortar, was dissolved in 10.0 mL of purified water at 25 ± 1 °C. The solution was stirred on a magnetic stirrer at 100 rpm until complete dissolution of the sample. The prepared solution was transferred to a secondary container, into which electrode tips were immersed, ensuring they did not touch the walls or bottom. For devices without a temperature compensation system, the sample temperature was maintained at 20 ± 2 °C. Once the device readings stabilized, the pH value was recorded. The result was taken as the arithmetic mean of three parallel measurements, with a maximum allowed difference of 0.1 pH unit, and rounded to the first decimal place. The overall error interval was ±0.1 pH unit at a confidence probability of P = 95% [18,21,24].

#### 2.2.3. In Vitro Dissolution Study

The microneedle matrix was immersed in 10.0 mL of purified water and kept at 32 ± 1 °C using a thermostat (conditions approximating skin surface temperature). The study was conducted without stirring to simulate post-application conditions on the skin. At defined time intervals (1, 5, 10, 15, 20 min), the sample was carefully removed with tweezers, and the degree of needle structure preservation was evaluated. The complete dissolution time was defined as the moment when none of the needles retained their original conical shape [25].

#### 2.2.4. Dissolution Uniformity Assessment

The same approach was used to evaluate dissolution uniformity. After 5 min of immersion in purified water at 32 °C, the microneedle matrix was removed, and the number of needles that had fully lost shape, partially retained structure, or remained unchanged was visually counted. Observations were conducted under standard laboratory lighting, using a magnifying glass (×5) when necessary.

Calculation was performed using Formula (1) [24]:

(1) $$\mathrm{Dissolution},\%=\frac{\mathrm{Number}\;\mathrm{of}\;\mathrm{dissolved}\;\mathrm{needles}}{\mathrm{Total}\;\mathrm{number}\;\mathrm{of}\;\mathrm{needles}\;\mathrm{in}\;\mathrm{the}\;\mathrm{frame}}\cdot 100$$

#### 2.2.5. Penetration Testing

Preparation of model gel: A 15% gelatin solution was prepared in purified water. While still hot, the solution was poured into polymer molds to form plates 8–10 mm thick. The plates were cooled to room temperature and then stabilized at 4–6 °C for 12 h.

Test procedure: At room temperature, the microneedle matrix was positioned perpendicular to the surface of the gel, and a weight of 0.5 g was applied for 30 s. After removing the matrix, the gel was visually inspected. The number of perforations was determined by counting the holes on the gel surface.

Calculation was performed using Formula (2) [24]:

(2) $$\mathrm{Penetration}\;\mathrm{efficiency},\%=\frac{\mathrm{Number}\;\mathrm{of}\;\mathrm{visible}\;\mathrm{punctures}}{\mathrm{Total}\;\mathrm{number}\;\mathrm{of}\;\mathrm{needles}}\cdot 100$$

Additionally, the presence of broken or deformed needles was recorded after testing.

Evaluation of microneedle penetration using the Parafilm M stacking method: The penetration capability of the microneedle systems was additionally evaluated using the Parafilm M stacking method, which is widely applied as a standardized in vitro model to simulate the mechanical resistance of skin. Parafilm M sheets were used as a model membrane and cut into identical rectangular segments, which were stacked to form an 8-layer assembly. The thickness of a single layer was approximately 100–150 μm, allowing simulation of progressively increasing mechanical resistance similar to that of skin layers.

Prior to testing, the layers were carefully aligned and fixed to prevent displacement during compression. The microneedle array was placed perpendicular to the surface of the multilayer Parafilm M stack, and a controlled force was applied for 30–60 s to ensure full contact between the microneedles and the membrane surface. After application, the layers were carefully separated and visually inspected for perforations. To enhance detection of microchannels, each layer was stained with a 1% (*w*/*v*) methylene blue solution, followed by gentle rinsing with distilled water to remove excess dye.

Penetration depth was determined as the number of pierced Parafilm layers, while penetration efficiency was calculated as the percentage of microneedles that penetrated at least the first layer relative to the total number of microneedles in the array.

The average penetration depth was defined as the mean number of fully or partially perforated layers for each formulation. All experiments were performed in triplicate (*n* = 3) [26].

#### 2.2.6. Mechanical Testing

The mechanical properties of the microneedles were determined using an axial compression test performed with a texture analyzer operating in tension–compression mode, equipped with a 50 N load cell (resolution 0.001 N, accuracy ± 0.1% of full scale).

Microneedle arrays were fixed on a rigid horizontal platform using double-sided adhesive tape to prevent displacement during measurement. The load was applied using a flat metallic indenter positioned perpendicular to the surface of the microneedle array.

The tests were conducted under the following conditions: pre-test speed, 0.5 mm/s; test speed, 0.2 mm/s; post-test speed, 0.5 mm/s; trigger force, 0.01 N; and compression distance, 0.6 mm.

The instrument operated within a speed range of 0.1–10 mm/s, enabling controlled loading conditions. The selected test speed minimized the influence of the viscoelastic properties of the polymer matrix. During the test, force–displacement curves were recorded continuously.

The failure force was defined as the maximum force prior to a sudden drop or change in the slope of the curve, indicating deformation or fracture of the microneedles.

Measurements were conducted at room temperature (22 ± 2 °C). For each sample, three independent measurements were performed (*n* = 3).

After testing, a visual assessment of microneedle deformation and structural integrity was additionally performed [27].

#### 2.2.7. Microscopy Analysis

For microscopic examinations, a Granum microscope (Granum Laboratory Ltd., Kharkiv, Ukraine) equipped with a Toupcam UCMOS video camera (Hangzhou ToupTek Photonics Co., Hangzhou, China) was used. Micrographs were captured and processed using ToupView 4.10 software from ToupTek (Hangzhou ToupTek Photonics Co., Hangzhou, China).

#### 2.2.8. Statistical Analysis

All experiments were conducted in at least three independent series. Statistical analysis was performed on six samples (*n* = 6). Data are presented as mean ± standard deviation (SD). Group comparisons were conducted using one-way ANOVA. Differences were considered statistically significant at *p* < 0.05. Prior to performing ANOVA, the normality of the data distribution in each group was assessed using the Shapiro–Wilk test, and the homogeneity of variances was evaluated using Levene’s test. Results were processed using Excel 2021 and StatisticKingdom software (online version) [18].

## 3. Results

Solutions for the microneedle matrix were prepared by dissolving the required amount of polymer in purified water using a magnetic stirrer at room temperature at 100 rpm (increasing the speed leads to the formation of a large number of air bubbles).

**Figure 3:**
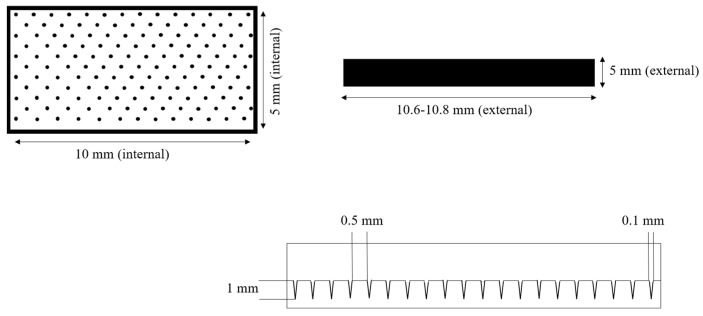
Schematic representation of a 3D-printed mold for microneedle casting.

**Figure 4:**
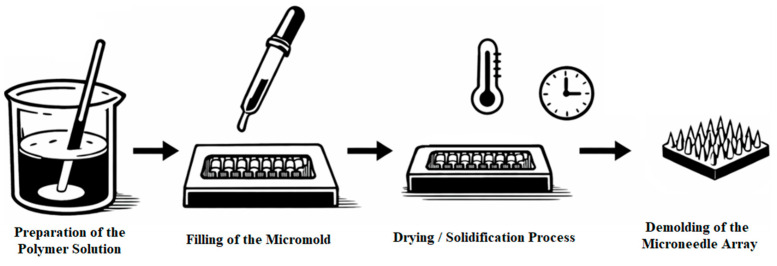
Workflow for microneedle fabrication using the casting method.

The solution was then transferred into molds (Figure 3) made from polymer Standard Resin using 3D printing on an ELEGOO Saturn 3 printer. Drying was conducted at room temperature for 1–2 days under controlled relative humidity not exceeding 60% (Figure 4) [23].

**Figure 5:**
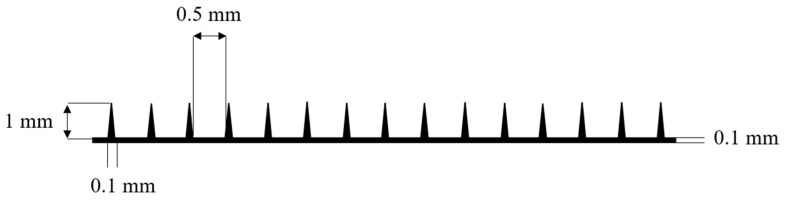
Schematic representation of the master mold.

**Figure 6:**
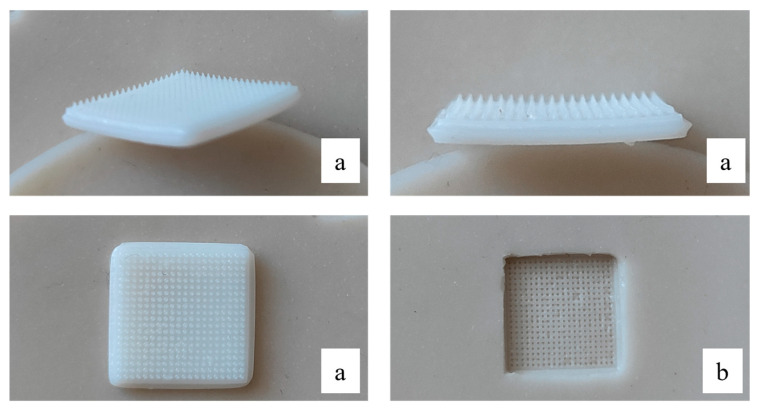
Master mold (**a**) and silicone mold (**b**).

In the subsequent stages of the study, a silicone mold (Silikony SK-760, Shenzhen, China, catalyst—CAT-303, Shenzhen, China) was used for microneedle casting, which we produced from a 3D-printed master mold with conical microneedles (Figure 5 and Figure 6). The master molds for microneedle fabrication were produced using masked stereolithography (MSLA) 3D printing technology (ELEGOO Saturn 3, Shenzhen, China). The digital model of the microneedle array was designed using CAD software 2022 and exported in STL format. Printing was performed using a photopolymer resin (Standard Resin, ELEGOO, Shenzhen, China) under a layer thickness of 0.05 mm. After printing, the molds were washed in isopropyl alcohol to remove uncured resin residues, and then post-cured under UV light to ensure complete polymerization and mechanical stability.

The printed master mold was subsequently used for fabrication of silicone negative molds (Silikony SK-760, China; catalyst CAT-303, China), which were prepared by casting and curing at room temperature according to the manufacturer’s instructions. The resulting silicone molds were used for microneedle casting.

The final microneedle geometry was measured after fabrication and drying, as significant shrinkage occurred during solvent evaporation. The obtained microneedle arrays had a size of 1 × 1 cm and consisted of approximately 400 needles/cm^2^. The average microneedle height was 550–700 µm, with a base diameter of 70–100 µm. The tip radius of curvature was estimated to be 5–15 µm. These values were lower than the master mold dimensions due to polymer shrinkage during drying and solidification. The resulting geometry was sufficient to ensure effective penetration and mechanical stability of the microneedle arrays.

The composition of polymer bases for microneedle matrix fabrication is presented in Table 1.

The total solid content of polymeric formulations ranged from 2% to 20% (*w*/*v*). These concentrations were selected based on preliminary screening experiments to cover low, medium, and high polymer loading conditions to evaluate their influence on mechanical strength and dissolution behavior.

We deliberately used polymers that are already well known and studied in this field, as we believe that established formulations can be improved. In particular, this includes achieving optimal strength, permeability, and solubility at lower polymer concentrations.

After removal from 3D-printed molds, most samples were deformed or had broken needles, so it was decided to switch to silicone molds for casting.

The casting method and mold material significantly affected the final morphology and mechanical integrity of the microneedles. In particular, silicone molds provided more uniform cavity filling and facilitated easier demolding, resulting in reduced tip deformation and improved structural reproducibility compared to rigid 3D-printed molds. Improved needle geometry directly influenced penetration performance, since mechanically stable and sharply formed microneedles demonstrated higher insertion efficiency in both gelatin and Parafilm M models.

Samples obtained from silicone molds exhibited the following characteristics:

Sample No. 1 (2% PVP K-30): Insufficient mechanical strength, while some needles had blunted tips and slight deformation after removal from the mold.

Sample No. 2 (5% PVP K-30): Improved needle geometry and sharper tips, though isolated deformations remained.

Sample No. 3 (10% PVP K-30): Uniform formation, clear conical tips, and no visible defects.

For methylcellulose-based samples, a concentration-dependent structural stability was observed:

Sample No. 4 (5%): Slight bending of individual needles.

Sample No. 5 (10%): Correct geometry and uniform structure.

Sample No. 6 (15%): Mechanically stable needles, but microcracks were noted at the base.

Sodium alginate-based microneedles demonstrated moderate shape-forming ability:

Sample No. 7 (5%): Partial tip deformation was observed.

Samples No. 8 (10%) and No. 9 (15%): Correct geometry; however, sample No. 9 showed increased brittleness.

Combined systems (Samples No. 10–15) showed the best morphological uniformity:

Sample No. 12 (5% PVP K-30 with 10% sodium alginate): Clear needle shape without defects.

Samples containing hyaluronic acid (No. 11, 14, 15) had a smoother surface without structural damage.

After penetration and dissolution tests, critical matrix failures were not detected in most samples, except for No. 1 and No. 7, where isolated broken needles were observed. Results of other analyses are presented in Table 2.

The results obtained using the Parafilm M stacking method were in good agreement with the trends observed in the gelatin-based model. In particular, a clear dependence was observed between the polymer matrix composition and the penetration depth of the microneedles. Formulations with lower polymer content exhibited limited ability to penetrate multiple Parafilm layers, which is consistent with their reduced mechanical stability observed in the gelatin penetration tests. In contrast, increasing the polymer concentration resulted in an improvement in both penetration depth in the Parafilm model and the formation of well-defined microchannels.

The best performance was observed for composite polymer systems, which enabled stable penetration through a greater number of Parafilm layers. This finding correlates with the high penetration efficiency obtained in the gelatin model (up to 96%), indicating consistency between the two independent penetration assessment approaches.

Overall, a positive correlation was established between the gelatin model and the Parafilm M stacking test, confirming the reliability of the obtained data. These results further suggest that composite polymer formulations are the most promising in terms of mechanical performance and their ability to form stable and reproducible microchannels in barrier-mimicking membranes.

The mechanical stability of the microneedle arrays varied over a wide range depending on the composition of the polymer matrix. It was found that formulations with a low polymer content exhibited limited mechanical strength, where initial deformation was observed even at relatively low loads, characteristic of soft polymer systems. According to the literature data on dissolving polymeric microneedles, the critical failure load typically ranges from 0.05 to 0.15 N/needle for weakly structured systems, and from 0.25 to 0.60 N/needle for optimized formulations capable of effectively penetrating the stratum corneum.

In the present study, low-concentration samples exhibited mechanical behavior corresponding to the lower limit of this range, whereas increasing the polymer content resulted in a shift toward mechanically stronger structures. Medium- and high-concentration systems demonstrated stable behavior under loads sufficient to induce skin penetration without critical loss of microneedle geometry.

The highest mechanical stability was observed for composite polymer systems, which are characterized by the formation of a denser three-dimensional polymer network. These systems typically achieve a mechanical strength level of approximately 0.35–0.65 N/needle, which is sufficient for reliable penetration through the human stratum corneum. The observed mechanical trends correlate well with the penetration test results, where insertion efficiency reached 42–96%, confirming the critical role of mechanical integrity in the functional performance of microneedle systems.

**Figure 7:**
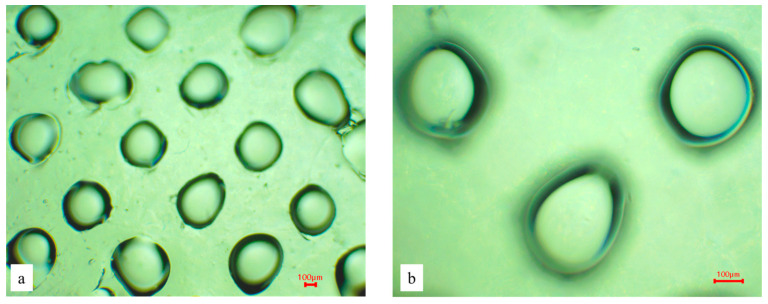
Microphotographs of sample No. 12; 4× (**a**), 10× (**b**).

It was found that increasing the polymer concentration enhances mechanical strength and penetration efficiency, but it also prolongs dissolution time. Combined formulations demonstrate an optimal balance between penetration and dissolution. The most optimal composition in terms of overall performance is sample No. 12 (5% PVP K-30 with 10% sodium alginate). The addition of hyaluronic acid to the base improved solubility and penetration efficiency of the microneedles, making it a promising component for further research. Sample No. 12, consisting of 5% polyvinylpyrrolidone and 10% sodium alginate, showed the best results both visually and according to the test results. Statistical analysis demonstrated significant differences between the studied samples (*p* < 0.05). In particular, sample No. 12 showed a statistically significantly higher penetration efficiency compared to low-polymer-content systems (Samples No. 1–5), confirming its suitability as the optimal formulation. A microscopic examination was conducted on this sample (Figure 7).

We obtained a horizontal projection of the sample (in the image, we can see the base of the microneedle near the surface of the scaffold), which confirms the size and integrity of the fabricated microneedle scaffold.

## 4. Discussion

The optimization of the microneedle composition in this study was based on a comparative experimental approach. Although a formal Design of Experiments (DoE) methodology was not applied, the obtained results allowed the identification of key relationships between the composition of the polymer matrix and the functional properties of the systems. In future studies, DoE approaches (particularly factorial design or response surface methodology) are planned to enable a more systematic optimization of the formulation.

The obtained results confirm that the morphological and functional characteristics of microneedle matrices depend on both the concentration and composition of the polymer matrix. With increasing polymer content, needle formation and mechanical strength improve, which is reflected in higher penetration efficiency into the gelatin model gel. Low concentrations provide rapid dissolution but are accompanied by reduced mechanical strength. Increasing the polymer concentration to 10–15% allowed the formation of strong, structured matrices with improved penetration (up to 90–96%), consistent with the reported literature on the influence of polymer matrix density in microneedle rigidity [27].

Other studies on dissolvable microneedles based on polyvinylpyrrolidone, methylcellulose, or their combinations have shown that achieving high penetration efficiency (80–100%) often requires higher polymer concentrations or the use of high-molecular-weight fractions, which simultaneously prolongs complete dissolution [28,29,30]. Similar trends have been widely reported for dissolving microneedles fabricated by solvent casting micromolding using PVP, PVA, hyaluronic acid, alginate, and cellulose-derived polymers, where increasing polymer concentration improves mechanical strength and penetration ability at the expense of prolonged dissolution time [1,31,32,33,34]. Our results confirm this: homopolymer matrices showed lower penetration during faster dissolution [35,36,37]. However, combined formulations, especially polyvinylpyrrolidone with methylcellulose or sodium alginate, provide both high penetration efficiency (>90%) and moderate complete dissolution time (8–12 min). Compared to published data, where higher polymer concentrations or stiffer matrices are needed to achieve similar penetration, our systems achieve equivalent mechanical properties with shorter dissolution times.

It was also found that the inclusion of hyaluronic acid improves structural uniformity while maintaining relatively fast dissolution and enhanced penetration, consistent with other reports of its plasticizing and hydrophilic effects in microneedle systems [23,38]. The pH values of all studied samples were within ranges close to physiological, meeting the requirements for topical application.

Compared to other studies, which often report either excessively rapid dissolution with insufficient mechanical strength or high penetration due to slow matrix degradation, our results demonstrate a more balanced quality profile. Combined polymer systems provide an optimal penetration/dissolution ratio without the need for significantly higher polymer concentrations, which is an advantage of the proposed approach in developing effective microneedle drug delivery systems.

The obtained results indicate the presence of a synergistic interaction between the components of the polymer matrix. In addition, the casting approach itself played an important role in the quality of the fabricated microneedles. The transition from rigid 3D-printed molds to elastic silicone molds improved the reproducibility of needle formation and reduced structural defects during demolding. These factors contributed to enhanced mechanical stability and, consequently, improved skin penetration ability. Similar observations have been reported in previous studies, where mold elasticity and cavity replication accuracy were identified as critical parameters affecting microneedle performance. In particular, the combination of polyvinylpyrrolidone and sodium alginate likely promotes the formation of a physically cross-linked structure through hydrogen bonding, which provides a simultaneous increase in mechanical strength and controlled swelling.

An important aspect is achieving a balance between penetration capability and dissolution rate. In contrast to approaches focused on maximizing a single parameter, the proposed compositions exhibit a balanced property profile, which is critically important for the practical application of microneedle systems.

Finally, for a more comprehensive understanding of the position of the developed systems within the broader context of microneedle technologies, it is appropriate to consider the main classes of materials used for their fabrication. Such comparative analysis clearly highlights the advantages of dissolving polymer matrices, which combine high biocompatibility, controlled dissolution, and a low risk of tissue injury, in comparison with other material-based approaches (Table A1).

## 5. Conclusions

The study established that the composition and concentration of polymers in the matrix affect the morphological and functional properties of microneedle matrices. Increasing polymer content improves mechanical strength and penetration efficiency but also increases complete dissolution time. Combined systems demonstrate a more balanced profile compared to homopolymer formulations, providing sufficient mechanical stability along with optimal dissolution time.

Based on the obtained data, a combination of 5% aqueous polyvinylpyrrolidone with 10% sodium alginate was selected as the optimal base for microneedle matrix fabrication using the casting method. The inclusion of hyaluronic acid is promising for enhancing structural uniformity and maintaining rapid dissolution. Further research should focus on ex vivo penetration studies, release kinetics of active pharmaceutical ingredients, and the biocompatibility and safety of the materials.

At the next stages of the study, tests are planned that will allow a more detailed investigation of the properties of the obtained samples that showed the best results at this stage. In particular, these include ex vivo skin penetration experiments.

## Figures and Tables

**Table 1:** Composition of polymer bases, %.

No.	PVP K-30	Methylcellulose	Sodium Alginate	Hyaluronic Acid	Purified Water
1	2.0	-	-	-	to 100
2	5.0	-	-	-	to 100
3	10.0	-	-	-	to 100
4	-	5.0	-	-	to 100
5	-	10.0	-	-	to 100
6	-	15.0	-	-	to 100
7	-	-	5.0	-	to 100
8	-	-	10.0	-	to 100
9	-	-	15.0	-	to 100
10	5.0	10.0	-	-	to 100
11	-	-	10.0	5.0	to 100
12	5.0	-	10.0	-	to 100
13	-	10.0	10.0	-	to 100
14	5.0	-	-	5.0	to 100
15	-	10.0	-	5.0	to 100

**Table 2:** Quality control results of microneedle matrices, *n* = 6, P = 95%.

No.	pH	Dissolution Time (min)	Dissolution Uniformity (%)	Penetration Efficiency (%)
1	6.2 ± 0.05	2.0 ± 0.3	97 ± 3	42 ± 6
2	6.3 ± 0.04	4.1 ± 0.4	90 ± 4	68 ± 5
3	6.4 ± 0.03	7.5 ± 0.6	72 ± 5	91 ± 4
4	6.8 ± 0.06	8.2 ± 0.7	70 ± 6	58 ± 5
5	6.9 ± 0.05	12.4 ± 0.8	52 ± 5	83 ± 4
6	7.0 ± 0.05	>20	35 ± 4	94 ± 3
7	7.2 ± 0.04	9.1 ± 0.6	60 ± 6	49 ± 6
8	7.3 ± 0.05	14.8 ± 1.0	45 ± 5	71 ± 5
9	7.4 ± 0.04	>20	28 ± 4	88 ± 4
10	6.6 ± 0.05	10.4 ± 0.6	68 ± 6	92 ± 4
11	6.3 ± 0.04	12.6 ± 0.7	50 ± 5	79 ± 4
12	6.8 ± 0.05	8.5 ± 0.5	78 ± 5	96 ± 3
13	6.9 ± 0.04	14.2 ± 0.8	48 ± 5	85 ± 4
14	5.9 ± 0.06	6.8 ± 0.5	82 ± 4	87 ± 5
15	6.0 ± 0.05	9.3 ± 0.6	65 ± 5	84 ± 4

## Data Availability

The original contributions presented in this study are included in the article. Further inquiries can be directed to the corresponding authors.

## Appendix A

**Table A1:** Comparison of different microneedle materials, properties, and application types.

Microneedle Type	Material	Mechanical Strength	Dissolution/Degradation Behavior	Biocompatibility	Risk of Tissue Damage	Main Application Areas
Metallic microneedles	Stainless steel, titanium	Very high	Non-dissolving	High (biologically inert)	Medium (risk of microtrauma during removal)	Vaccination, diagnostic sensors, delivery of infrequent or high-dose drug administrations
Solid polymer microneedles	Polymethyl methacrylate, polycarbonate, polyethylene terephthalate	High	Non-dissolving	High	Low	Reusable patch systems, coated transdermal drug delivery systems
Dissolving polymer microneedles	Polyvinylpyrrolidone, hyaluronic acid, poly(lactic-co-glycolic acid), sodium alginate	Medium–high (composition-dependent)	Complete dissolution in biological fluids	Very high	Very low	Transdermal delivery of vaccines, proteins, peptides, insulin, vitamins, antimicrobial agents
Hybrid microneedle systems	Metal core with polymer coating or drug-loaded coating (metal-core polymer-coated microneedles)	Very high	Partial or controlled release of coating	High	Medium	Vaccination, controlled insulin delivery, hormone therapy, diagnostic systems with sensing layers
